# Isolated Gall Bladder Perforation in A Tertiary Care Hospital in Eastern Nepal: A Descriptive Cross-sectional Study

**DOI:** 10.31729/jnma.5267

**Published:** 2020-12-31

**Authors:** Brikh Raj Joshi, Swotantra Gautam, Saroj Adhikari Yadav, Sushil Dhakal, Rasmita Thapaliya, Rakesh Kumar Gupta

**Affiliations:** 1 Department of Surgery, Lumbini Provincial Hospital, Butwal, Rupendehi, Nepal; 2 B. P. Koirala Institute of Health Sciences, Dharan, Nepal; 3 Patan Academy of Health Sciences, Patan, Nepal; 4 Department of Pathology, Maya Metro Hospital, Dhangadi, Nepal; 5 Department of Nursing, Sanjjevani College of Medical Sciences, Rupendehi, Nepal; 6 Department of Surgery, B. P. Koirala Institute of Health Sciences, Dharan, Nepal

**Keywords:** *cholecystitis*, *classification*, *gallbladder*, *perforation*

## Abstract

**Introduction:**

Cholelithiasisis is a common surgical problem worldwide. Gall bladder perforation is a rare life-threatening complication with considerable mortality. This study aims to find the etiology, demography, type of perforation, and outcome of gall bladder perforation.

**Methods:**

This descriptive cross-sectional study was done on patients above 18 years of age visiting department of surgery of B. P. Koirala Institute of Health Sciences (BPKIHS) who were diagnosed with isolated gall bladder perforation. The study was done from 1st January 2006 till 30 December 2016. Ethical approval was obtained from the Institutional Research Committee (reference number. 34/074/075). The convenient sampling method was used. Data were entered in excel sheets and analyzed.

**Results:**

Out of 49 patients included in the study, 28 (57.14%) were females and the commonest age group was 36 to 50 years 22 (44.9%) followed by 51 to 65 years 16 (32.6%). Most of the patients presented in emergency with pain in their abdomen. Diabetes mellitus was the commonest co-morbidity present in 10 (20.41%) patients. Operative management was done in 45 (91.84%) of the patient and conservative management in 4 (8.16%). After surgery of 45 patients, 43 (95.56%) improved and 2 (4.44%) expired. The most common type of perforation was Niemeier Type I in 21 (46.67%) followed by Type III 14 (31.11%). The most common histopathological diagnosis was acute cholecystitis 20 (44.44%).

**Conclusions:**

Isolated gall bladder perforation is not an uncommon complication. The most common etiological factor was acute cholecystitis with a slight female predominance. Most of the patients needed surgical intervention and they had good outcomes when diagnosed and managed on time.

## INTRODUCTION

Gallbladder perforation (GBP) is a rare life-threatening condition. Cholelithiasis is a common surgical problem worldwide. GBP is a rare complication of different gallbladder disease.^1^ Common etiological factors include cholecystitis, gall bladder carcinoma, traumatic perforation, etc. Most cases of GBP can only be diagnosed during surgery.^2^

Because of delays in diagnosis, there is high morbidity and mortality. Thus, GBP continues to be an important problem for surgeons.^3,4^ The mortality rate of GBP ranges from 12 to 42%.^2,5^ Niemeier in 1934, classified GBP into three types, viz, Type I-acute perforation into the free peritoneal cavity and generalized biliary peritonitis, Type II-subacute perforation with abscess formation and localized peritonitis; and Type III- chronic perforation with fistula formation between the gallbladder and another viscus.^6^

After an extensive literature search, we found scarcity in studies regarding the GBP. This study aims to find out the etiology, patient demography, type of perforation, and outcome of GBP.

## METHODS

This study was conducted in the department of surgery of B. P. Koirala Institute of Health Sciences (BPKIHS), Dharan, Nepal. Record sheets of the patients who had isolated GBP were retrieved. The study included all the patients above 18 years of age, admitted as a case of isolated GBP from 1st January 2006 till 30 December 2016 (11 years). Ethical approval was taken from the Institutional Research Committee of BPKIHS (Ref No. Acad. 34/074/075). Records with grossly incomplete data were excluded from the study. Convenient sampling was done.

Sample size was calculated as,

$$\begin{array}{ll} \text{n} & \text{= }{\text{Z}}^{\text{2}}\times \text{p}\times (\text{1}-\text{p)}/{\text{e}}^{\text{2}}\\ & \text{= }{\left(1.96\right)}^{\text{2}}\times 0.5\times (1-0.5)/{(\text{0.14)}}^{\text{2}}\\ & \text{= }49 \end{array}$$

where,

n = Sample size
Z = 1.96 at 95% Confidence Interval
p = population proportion, 50%
e = margin of error, 14%

Thus a total of 49 samples were included in this study. Patients presenting in the emergency with features of peritonitis (generalized) were planned for emergency exploratory laparotomy after all the preoperative investigation. Written consent was taken from the patient and patient relative. After general anesthesia and endotracheal intubation prepping and draping of the operative site were done. A generous midline incision was made and rectus sheath was incised with the help of electric cautery. The peritoneum was opened, bilious fluid was sucked and peritoneal lavage was done with normal saline. Intraoperative findings were noted. Either anterograde or retrograde cholecystectomy was performed according to intraoperative findings. Hemostasis was secured and an intraabdominal drainage tube of 28 Fz was kept in the right subhepatic space. Rectus sheath was closed with polypropylene number 1 suture and skin was closed with nylon 3-0 sutures.

In elective cases, after general anesthesia and endotracheal intubation prepping and draping were done. Pneumoperitoneum was created by open (Hasson's) technique. The thirty-degree telescope was inserted and then three working ports were made (10 mm port 3 to 5 cm below xiphisternum, 5 mm port 2 to 3 cm below right subcostal margin in midclavicular line and5 mm port in anterior axillary line 3 to 4 cm below the costal margin). Then the patient was kept in a reverse Trendelenburg position and fifteen degrees left tilt. Gallbladder fundus was retracted toward the right shoulder and intraoperative findings were noted. If there was evidence of fistula between gallbladder and gastrointestinal tract (cholecystogastric, cholecystoduodenal, or cholecystocolic fistula) then the procedure was converted to open cholecystectomy. Right subcostal incision (Kocher's incision) was made. Muscles were divided and the peritoneum was opened. Intraoperative findings were noted and management was done according to intraoperative findings. Generally, the fistulous tract was excised and the hollow viscus defect was closed with polyglactin suture 3-0 round body needle.

Data were collected in predesigned proforma regarding the demographic profile, clinical presentation, general physical examination, per abdominal examination, positive findings on systemic examinations, investigations (Ultrasonography (USG), X-ray abdomen, contrast-enhanced computed tomography (CECT) abdomen), type of management done (conservative or surgical and type of surgery) and histopathological reports. Data were entered in an excel sheet and converted into Statistical Package for the Social Sciences software (SPSS) version 11.5 for statistical analysis. The data were presented in number and percentage.

## RESULTS

**Figure 1:**
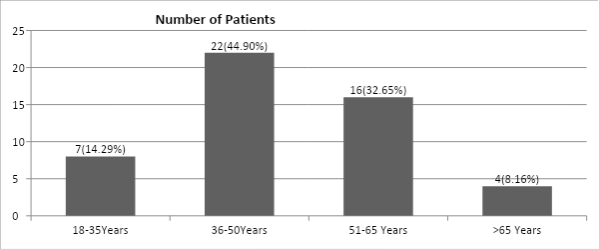
Age-wise distribution.

Out of 49 patients, 28 (57.14%) were females and 21 (42.86%) were males. The most common age group affected was 36 to 50 years of age 22 (44.90%) followed by 51 to 65 years of age group 16 (32.65%) (Figure 1).

Among them, 31 (63.27%) patients presented in the emergency and 18 (36.73%) in OPD (Outpatient Department). All the patients had abdominal pain. Fever was present in 25 (51.02%). Only 5 (10%) patients had a history of abdominal trauma. Most of the patients 32 (65.31%) had no comorbidities. Comorbidities associated were diabetes mellitus in 10 (20.41%), hypertension 4 (8.16%), steroid use 2 (4.08%), and chronic obstructive pulmonary disease (COPD) 1 (2.04%). Abdominal ultrasonography was done in 47 (95.92%) of the patient but the diagnosis of GBP was made by USG in only 3 (6.12%) patients (Table 1).

**Table 1:** History and physical examination findings.

Findings	Variables	n (%)
Presented at	OPD	18 (36.73)
	Emergency	31 (63.27)
Abdominal Pain	Localized	28 (57)
	Generalized	21 (43)
Fever	Yes	25 (51)
	No	24 (49)
Abdominal trauma	Yes	5 (10)
	No	44 (90)
	Tenderness	41 (37.3)
	Guarding	30 (27.3)
Abdominal findings	Rebound tenderness	30 (27.3)
	Normal	9 (8.2)
	No Comorbidities	32 (65.31)
	Diabetes Mellitus	10 (20.41)
Comorbidities	Hypertension	4 (8.16)
	Steroid Use	2 (4.08)
	COPD	1 (2.04)

The majority of patients, i.e. 45 (91.84%) received operative treatment and 4 (8.16%) were managed by conservative treatment. Total mortality was 6 (12.24%). All four patients managed conservatively. Among 45 surgically managed patients, 43 (95.56%) improved and 2 (4.44%) expired (Table 2).

**Table 2:** Treatment and outcome (n = 49).

Management	Frequency n (%)	Outcome	
		Improved n (%)	Expired n (%)
Conservative	4 (8.16)	0	4 (100)
Operative	45 (91.84)	43 (95.56)	2 (4.44)
Total	49 (100)	43 (87.76)	6 (12.24)

Among the 45 patients treated operatively, the most common site of GBP was fundus 33 (73.33%). 21 (46.47%) of patients had Niemeier Type I, 10 (22.22%) had Type II and 14 (31.11%) had Niemeier Type III GBP. According to histopathological report, the most common etiology for GBP was acute cholecystitis 20 (44.44%), followed by acute on chronic cholecystitis 12 (26.67%), chronic cholecystitis 9 (20%), adenocarcinoma of the gallbladder 3 (6.67%), and Salmonella typhi infection in 1 (2.22%) (Table 3).

**Table 3:** Site and type of perforation of 45 operatively managed patients (n = 45).

Observations	Variable	Frequency n (%)
Site of perforation	Body	12 (26.67)
	Fundus	33 (73.33)
Type of Perforation	Niemeier type I	21 (46.67)
	Niemeier type II	10 (22.22)
	Niemeier type III	14 (31.11)
Histopathological Diagnosis	Acute Cholecystitis	20 (44.44)
	Acute on Chronic Cholecystitis	12 (26.67)
	Chronic Cholecystitis	9 (20)
	Adenocarcinoma of GB	3 (6.67)
	Salmonella Typhi	1 (2.22)

## DISCUSSION

In this study, we found that GBP was slightly more predominant in females 57.14% than males 42.86%. But a study done by Derici, Stefanidis, and Ergul found that males were having more perforation than females (62.5%, 76.7%, and 54.1% respectively).^6–8^ This contradiction may be because of more prevalence of gall stone in females in our set up and so the complications.

Abdominal ultrasonography was done in 95.92% of the patients but the only diagnosis was made by USG in only 6.12% of the patients. This may be because of the low-resolution ultrasound scanner. Sood, et al. reported that the sonographic hole sign is the only reliable sign of gallbladder perforation and was only visible by a high-resolution ultrasound scanner device in 70% of patients.^2^ In our study only a few patients underwent computed tomography (CT) scan of the abdomen due to either unavailability or unaffordability of abdominal CT in an emergency.

As much as 91.84% of patients received operative treatment in the form of open cholecystectomy or laparotomy with cholecystectomy and 8.16% were managed conservatively. Conservative treatment was done for patients who were unfit for surgery. Conservative treatment was done by percutaneous abdominal drain placement in subhepatic space, fluid and electrolyte supplement, and intravenous antibiotics (both aerobic and anaerobic coverage). All the patients under conservative treatment expired probably due to multiple comorbid conditions like diabetes mellitus, chronic respiratory disease, sepsis, septic shock, and multiple organ dysfunctions.

Among surgically 45 managed patients, most of the patients were managed with open operation rather than laparoscopically. This is probably because of surgeon preference and due to technical difficulties for the repair of a fistula between the gallbladder and hollow viscuslaparoscopically.

Mortality in our study was 12.24%, however, there were only two mortalities among 45 operated cases and all four patients under conservative treatment died. Anderson BB also reported that ultimately cholecystectomy was mandatory for the definitive treatment of GBP.^9^ Due to a lack of imaging modality, the mortality was as high as 40% in the past. But now it came down to 10 to 12%.^11^ Glenn and Moore have reported that the mortality rate in patients with gallbladder perforation was 42%.^5^ Whereas, other studies reported that the mortality rates have decreased to 12%-16% owing to the developments in anesthesiology and intensive care conditions.^7^ Higher percentage of mortality in our study was due to delay in diagnosis and treatment as well as the nature of the disease, comorbidities, and complications like sepsis, septic shock, and multi-organ dysfunctions.

In this study, the most common etiology was acute cholecystitis 44.44% followed by acute on chronic cholecystitis 26.67%, chronic cholecystitis 20%. Other studies too showed cholecystitis as the commonest etiology for GBP.^7,11–13^ The etiology was adenocarcinoma of the gallbladder in 6.66% in our study but adenocarcinoma is not reported as a common cause of GBP in other studies. ^6–8,11–13^

We found that the most common site of GBP was fundus 67.35% of the gall bladder because it is the most remote area concerning blood supply as described by Roslyn J.^10^ In this study we found that 46.67% of patients had Niemeier Type I, 22.22% had Type II and 31.11% had Type III GBP.

## CONCLUSIONS

Isolated GBP is not an uncommon complication in our tertiary care center. The most common etiological factor was acute cholecystitis with a slightly female predominance. Niemeier Type I was the commonest type of perforation. Most of the patients needed surgical intervention and had a good outcome when diagnosed and managed on time.
